# Koch’s Bacillus and Its Lessons

**Published:** 1883-04

**Authors:** 


### KOCH'S BACILLUS AND ITS LESSONS.

The announcement by Koch of the discovery of a bacillus, to whose entrance into the system specific tuberculosis is due, is likely to prove one of the most important in the history of medicine. It is true the existence of such a parasite has been quite strongly combatted by authority which commands respect, but a careful weighing of the evidence, *pro et con*, must, we think, satisfy the judicial mind that Koch has made out his case, and that the tubercle-bacillus is one of the facts of science. Regarded purely as a theory, it certainly furnishes a satisfactory explanation of the pathological conditions of which it is declared to be the immediate cause.

Koch's experiments, as reported, are exceedingly interesting as well as convincing, the latter property being due to the careful attention to detail and the elimination of every conceivable factor likely to vitiate results. Herein they differ from the generalizations of Pasteur. The latter, for instance, has not in every instance demonstrated the existence of the bacteria which he has assumed. Having isolated the specific cause of charbon, or splenic fever, in cattle and sheep, he assumes, but does not demonstrate, the existence of an analogous cause in such diseases as scarlatina, diphtheria, yellow fever, etc. It is true the grounds for such assumption are very conclusive, but it is not truly scientific to assume. Koch has pointed out that in employing the secretions found in dead, for introduction into living animals, with a view to reproducing the fatal disease, Pasteur has committed the error of introducing with his hypothetical bacillus septic material and the cadaveric alkaloids, which latter it is well known are of a very deadly nature. In his experiments he (Koch) has assumed nothing, but has clearly isolated the bacilli, and by their introduction into other animals has produced an affection which cannot be duplicated by inoculation with any other material, even though it be of a very septic nature. Pasteur, moreover, in selecting his animals, has not been careful to first demonstate that the animals experimented with have in every instance been susceptible to the specific disease sought to be propagated; and not being himself a physician, has not been qualified to determine whether the resulting affection has been the true disease, or pyæmia or septicæmia. Koch has, as we have said, avoided all these elements of error.

Granted that the tubercle-bacillus is a fact, what are we to do with it? On the answer to this question rests the future treatment of consumption, prophylactic and curative, and the ingenuity of man will be directed both to the prevention of its entrance into the system and to its destruction in cases in which it has inadvertently, or in spite of prophylactic measures, found an entrance. When the means to these ends have been discovered, consumption will occur only as a result of negligence, and will prove fatal only as a result of improper or insufficient treatment. When these means shall have been discovered, medicine will have purged itself of the greatest of its opprobia.

Although the means of prophylaxis and cure, above referred to, are still veiled from our eyes (for we have faith in their existence), the discovery of the bacillus and of its nature has already directed an intelligent treatment of consumption. It is fair to assume that it, like the germs of other diseases, is more or less constantly present in every community, floating about, as it were, seeking an opportunity to enter into systems in which it may propagate and work its mischief. It follows, then, that something more than the bacillus is necessary to produce consumption; it must have an appropriate soil. That soil, as is the soil in which other germs of disease take root and bring forth fruit, some thirty fold and some sixty fold and some an hundred fold, is characterized by an absence of the power of vital resistance. We know that the local lesion is by no means the first symptom of the affection which develops into full-blown tuberculosis. Preceding this there are dyspeptic disturbances, loss of appetite, emaciation, and a lowering of the vital forces. While these may not properly be a part of the consumption, they are what prepare the soil for the growth of the germ. It is fair to assume that the air-passages of those brought in contact with tuberculous patients frequently contain bacilli, which, being met at the portals by the vital resistance furnished by the system in its average health, fail to effect an entrance, degenerate and are thrown off. It would follow, therefore, that the maintenance of the system at its full standard of vitality is the most effective prophylaxis against the ingress of the bacillus, which, when it gains admission, acts as a foreign body, and sets up the circumscribed inflammation which constitutes solidification, and which in turn breaking down causes the tuberculous cavity.

The discovery of the tubercle-bacillus antagonizes the theory of the hereditary nature of consumption *per se.* While in very many instances the disease appears to run in families, it will hereafter be more correct to say that the tendency to it is hereditary, or, rather, that the weakened power of resistance to the entrance of bacillus is transmitted.

The etiology of consumption being thus referred to a specific cause, the result must be to strew the shores with additional wrecks of theories of treatment. From Salisbury's rare beef to Churchill's hypophosphites the therapeutic measures are legion, for they are many, and these must all give way before the agent yet to be discovered, which shall have the power of entering the system, and engaging the bacillus in combat, lay it low. The treatment of consumption by means of an atmosphere holding germicides in suspension must, in the nature of the case, attract renewed attention through the discovery of Koch's bacillus. It would seem that in this direction lies the means which shall enable us to save the hundreds of thousands of lives which are annually lost from consumption. Many of the agents now employed will doubtless remain useful as means for combating symptoms, but the curative agent proper will be a germicide. Koch has covered himself with a fame scarcely secondary to that which surrounds the name of Jenner, but the blaze of glory and fame immortal awaits him who discovers the agent which shall ferret out the microscopic bacillus in its fastness in the tissue of the human lungs, and, giving it battle, shall destroy it, root and branch.—*Therapeutic Gazette.*

